# Anti-Proliferative Activity of Triterpenoids and Sterols Isolated from *Alstonia scholaris* against Non-Small-Cell Lung Carcinoma Cells

**DOI:** 10.3390/molecules22122119

**Published:** 2017-12-01

**Authors:** Chao-Min Wang, Kuei-Lin Yeh, Shang-Jie Tsai, Yun-Lian Jhan, Chang-Hung Chou

**Affiliations:** 1 Research Center for Biodiversity, China Medical University, Taichung 40402, Taiwan; wangchaomin@mail.cmu.edu.tw (C.-M.W.); csungjay@yahoo.com.tw (S.-J.T.); ah_giu@yahoo.com.tw (Y.-L.J.); 2 Department of Laboratory, Chang Bing Show Chwan Memorial Hospital, Changhua 500, Taiwan; seehome2001ya@yahoo.com.tw; 3 Department of Biological Science and Technology, China Medical University, Taichung 40402, Taiwan

**Keywords:** *Alstonia scholaris*, triterpenoid, sterol, non-small-cell lung carcinoma cells (NSCLC), ursolic acid, betulinic acid, betulin, 2β,3β,28-lup-20(29)-ene-triol

## Abstract

(1) Background: In China and South Asia, *Alstonia scholaris* (Apocynaceae) is an important medicinal plant that has been historically used in traditional ethnopharmacy to treat infectious diseases. Although various pharmacological activities have been reported, the anti-lung cancer components of *A. scholaris* have not yet been identified. The objective of this study is to evaluate the active components of the leaf extract of *A. scholaris*, and assess the anti-proliferation effects of isolated compounds against non-small-cell lung carcinoma cells; (2) Methods: NMR was used to identify the chemical constitutes isolated from the leaf extract of *A. scholaris*. The anti-proliferative activity of compounds against non-small-cell lung carcinoma cells was assessed by 3-(4,5-dimethylthiazol-2-yl)-2,5-diphenyltetrazolium bromide (MTT) assay; (3) Results: Eight triterpenoids and five sterols were isolated from the hexane portion of *A. scholaris*, and structurally identified as: (**1**) ursolic acid, (**2**) oleanolic acid, (**3**) betulinic acid, (**4**) betulin, (**5**) 2β,3β,28-lup-20(29)-ene-triol, (**6**) lupeol, (**7**) β-amyrin, (**8**) α-amyrin, (**9**) poriferasterol, (**10**) epicampesterol, (**11**) β-sitosterol, (**12**) 6β-hydroxy-4-stigmasten-3-one, and (**13**) ergosta-7,22-diene-3β,5α,6β-triol. Compound **5** was isolated from a plant source for the first time. In addition, compounds **9**, **10**, **12**, and **13** were also isolated from *A. scholaris* for the first time. Ursolic acid, betulinic acid, betulin, and 2β,3β,28-lup-20(29)-ene-triol showed anti-proliferative activity against NSCLC, with IC_50_ of 39.8, 40.1, 240.5 and 172.6 μM, respectively.; (4) Conclusion: These findings reflect that pentacyclic triterpenoids are the anti-lung cancer chemicals in *A. scholaris*. The ability of ursolic acid, betulinic acid, betulin, and 2β,3β,28-lup-20(29)-ene-triol to inhibit the proliferative activity of NSCLC can constitute a valuable group of therapeutic agents in the future.

## 1. Introduction

In the past few decades, non-small-cell lung cancer (NSCLC), one of the most commonly diagnosed malignancies, has been shown to be the leading cause of cancer-related mortality all over the world. In all lung cancer cases, 75% to 80% have been identified as non-small-cell lung cancer, while only 15% to 25% is small cell lung cancer (SCLC). It is noted that conventional treatment of either form of lung cancer is fairly ineffective [1]. Thus, the development of new therapeutic strategies against NSCLC is urgently needed. Previous studies have demonstrated that extracts from some herbal medicines have anti-lung cancer potential and can inhibit lung cancer cell proliferation [2,3,4,5]. Recently, many of the chemotherapeutic agents are medicinal plants or are derived from medicinal plants. Therefore, attention has been paid to investigate the natural, active ingredients from medicinal plants against lung cancer cell. 

The *Alstonia scholaris,* belonging to the family Apocynaceae, is widely distributed in the tropical regions of Africa and Asia [6]. It is a tropical evergreen tree native to South and Southeast Asia, and is called blackboard tree, or milkwood pine, commonly. Traditionally, the leaves of *A. scholaris* have been used in “Dai” ethnopharmacy to treat chronic respiratory diseases in China [7]. In Africa, Australia, India, Malaysia, the Philippines, and Thailand, *A. scholaris* are also used in traditional medicinal systems [7]. The extracts of *A. scholaris* possess a wide spectrum of pharmacological activities; as a result, the chemical constituents of *A. scholaris*, especially the alkaloids, have been extensively investigated [8,9,10,11,12,13]. The extracts of *A. scholaris* have been observed to possess anti-diabetic [14], anti-inflammatory [15], anti-tussive, anti-asthmatic, and expectorant activities [16]. Recently, the potential of *A. scholaris* on antimicrobial activity has been screened, and the potent chemical constitutes and their exact effective concentration have also been identified [17]. These findings reflect that the pleiotropic effects of ursolic acid against methicillin-resistant *Staphylococcus aureus* (MRSA) make it a promising antibacterial agent in pharmaceutical research [18]. Although the pharmacological usage of *A. scholaris* has been greatly investigated, the anti-proliferative activity against NSCLC is not clear. Therefore, the aim of this study was to further investigate the anti-proliferative constitutes from the leaf extracts of *A. scholaris* against NSCLC. It is suggested that these compounds might be a valuable group of therapeutic agents in NSCLC treatment in the future.

## 2. Results

### 2.1. Isolation and Identification of Triterpenoids from A. scholaris

**Figure 1:**
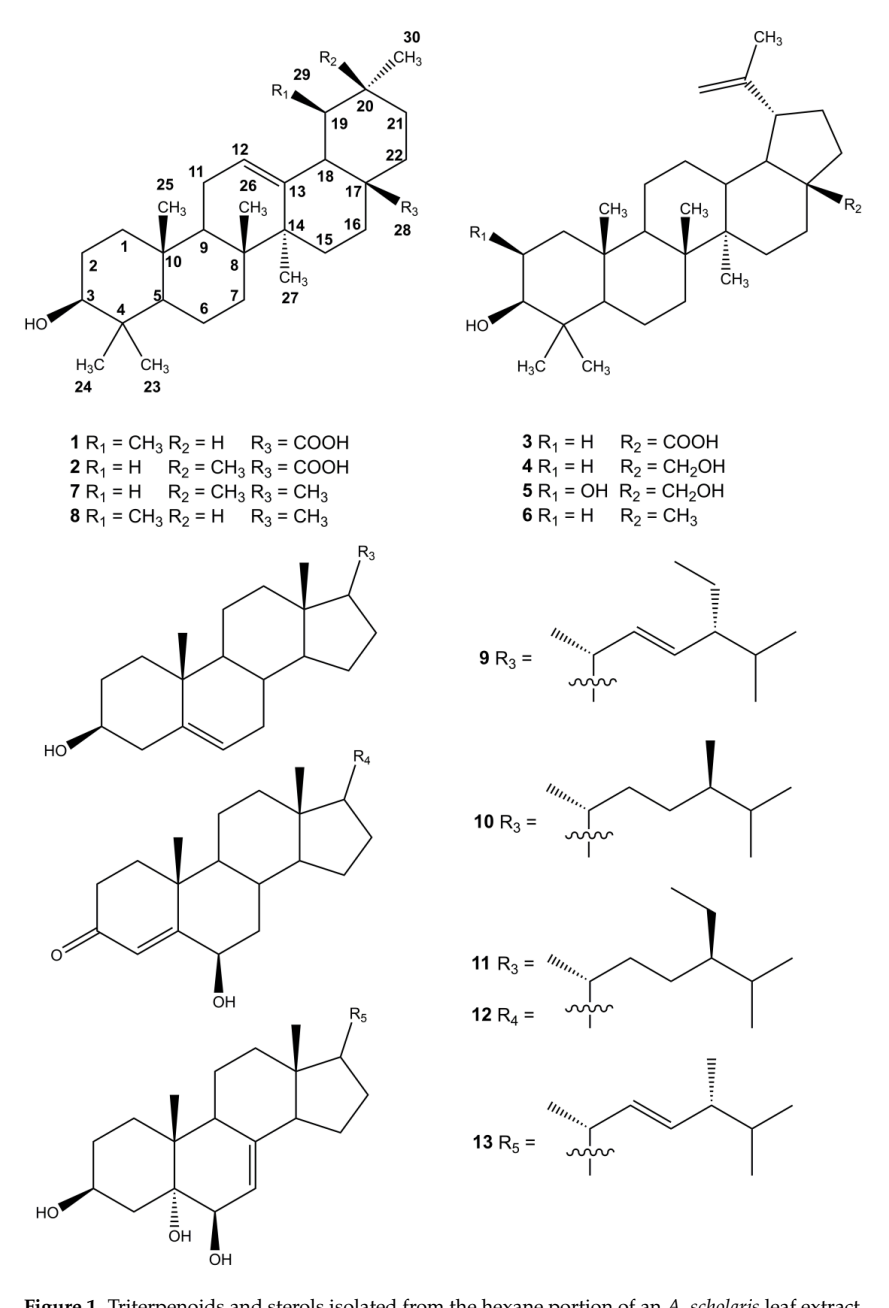
Triterpenoids and sterols isolated from the hexane portion of an *A. scholaris* leaf extract.

The anti-proliferative constitutes of the most effective fractions in the hexane portion (fraction Hex-4 to Hex-7) were isolated by using column chromatography to obtain 13 pure compounds: compound **1** (4.61 mg), **2** (4.47 mg), **3** (1.01 mg), **4** (3.01 mg), **5** (1.88 mg), **6** (4.0 mg), **7** (4.1 mg), **8** (2.74 mg), **9** (4.9 mg), **10** (2.59 mg), **11** (7.29 mg), **12** (3.15 mg), and **13** (16.15 mg). Purified compounds were subjected to spectroscopic identification by using ^1^H-NMR, ^13^C-NMR (Agilent Technologies DD2 600), and Mass (Bruker Daltonics Esquire HCT). All of the proton and carbon signals were assigned based on the ^1^H-^1^H correlation spectroscopy (COSY), distortionless enhancement by polarization transfer (DEPT) analysis, heteronuclear multiple-quantum correlation (HMQC), and heteronuclear multiple bond correlation (HMBC). The chemical structures of triterpenoids (**1**–**8**) and sterols (**9**–**13**) were illustrated in Figure 1. 

By comparing the NMR and mass (MS) data with previous reports, compounds isolated from the leaves of *A. scholaris* were identified as ursolic acid (**1**) [19], oleanolic acid (**2**) [19], betulinic acid (**3**) [20], betulin (**4**) [21], upeol (**6**) [21], β-amyrin (**7**) [22], α-amyrin (**8**) [23], poriferasterol (**9**) [24], epicampesterol (**10**) [25], β-sitosterol (**11**) [26], 6β-hydroxy-4-stigmasten-3-one (**12**) [27], and ergosta-7,22-diene-3β,5α,6β-triol (**13**) [28] (Figure 1), respectively.

Compound **5** was isolated from the hexane fraction AS-H-6-6-6-2 by HPLC. As shown in Table 1, The ^1^H-NMR spectrum (CDCl_3_, 600 MHz) revealed the presence of a pair of olefinic protons at δ 4.69 and δ 4.59 (each one H, brs), which is characteristic of an exocyclic methylene group; 6 singlet methyls at δ 0.99 (3H, s, Me-23), 0.98 (3H, s, Me-24), 1.14 (3H, s, Me-25), 1.04 (3H, s, Me-26), 0.97 (3H, s, Me-27), and 1.68 (3H, s, Me-30); and two carbinolic protons at δ 4.09 (dd, *J* = 3.6, 6.6 Hz, H-2) and 3.19 (d, *J* = 4.2 Hz, H-3), referring to the axial and α orientation. The ^13^C-NMR spectrum (CDCl_3_, 150 MHz) showed the presence of 30 carbons comprising six methyls, 11 methylenes, seven methines, and six quaternary carbons. There was a vinyl carbon signal at 109.6 ppm, the signal corresponding to methylene–methylidene at 150.4 ppm, and two carbon bound to the hydroxyl group at 78.4 and 71.1 ppm, respectively. All of the proton and carbon signals were assigned based on the ^1^HCOSY, DEPT analysis, HMQC, and HMBC. According to the data shown in Table 1, compound **5** was identified as 2β,3β,28-lup-20(29)-ene-triol, a compound that has been synthesized previously [29]. Compound **5** was isolated from a plant source for the first time.

### 2.2. Antiproliferation Activity of Triterpenoids and Steriols against NSCLC

**Figure 2:**
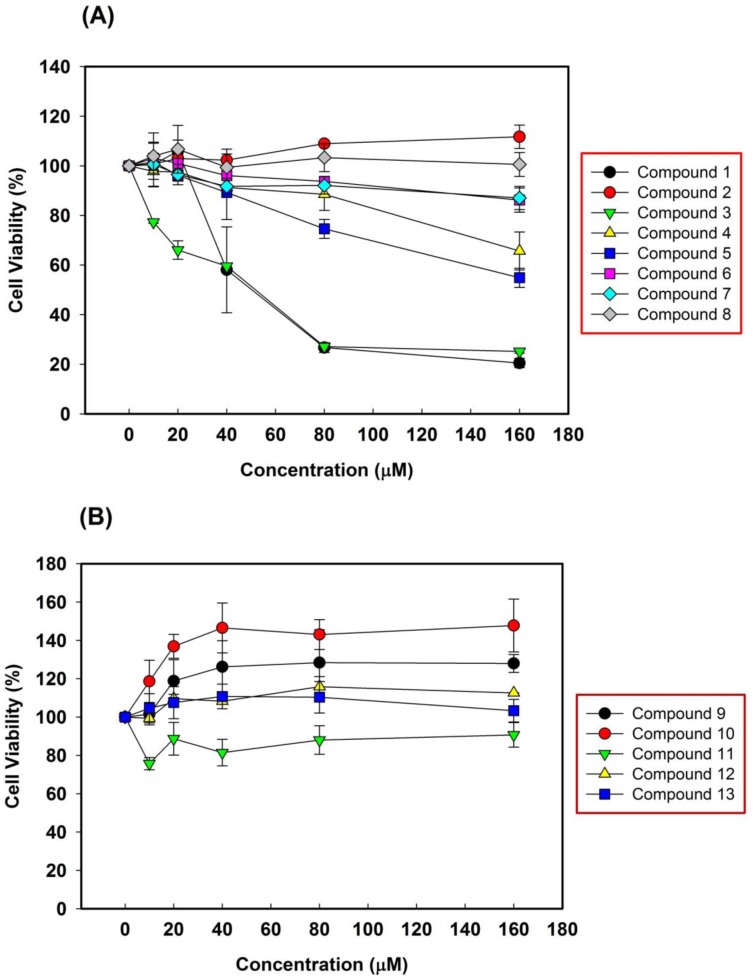
Anti-proliferation activities of triterpenoids (**A**) and sterols (**B**) from *A. scholaris* leaf extract.

To evaluate the anti-proliferative activities of isolated triterpenoids (Figure 2A) and sterols (Figure 2B) on NSCLC cells, A549 cells were treated with various concentrations of isolated compounds for 48 h. The cell viability was evaluated using the MTT assay. As shown in Figure 2A, the exposure of A549 cells to compounds **1**, **3**, **4** and **5** decreased cellular viability in a dose-dependent manner. In the treatment of sterols, only compound **11** showed an inhibitory effect on NSCLC cells, with a 20% decrease in cell viability. Interesting, compounds **9** and **10** showed no inhibiting effect on A549 cells, but did show an increasing proliferation effect (Figure 2B). These results showed that only triterpenoids exhibited efficient anti-proliferative effects on NSCLC cells in an *A. scholaris* leaf extract.

### 2.3. The Inhibitory Concentrations (IC_50_) of Triterpenoids and Steriols on NSCLC

The anti-proliferative activities of isolated triterpenoids (**1**–**8**) were determined by measuring the IC_50_ of NSCLC cells. As shown in Table 2, half of the isolated triterpenoids did not show any effect on NSCLC. Two triterpenoids, compounds **4** and **5**, displayed weak anti-NSCLC activities at IC_50_ values of 240.5 and 172.6 μM, respectively. In addition, at IC_50_ values of 39.8 and 40.1 μM, compounds **1** and **3** inhibited A549 cell growth.

## 3. Discussion

Triterpenoids are a group of structurally diverse metabolites that are often used as pharmaceuticals with various biological activities. Triterpenoids exist abundantly in *Alstonia* spp. and their proposed bioactivities include anti-HIV, anti-microbial, allelopathy, anti-tumor, and anti-cancer activities [17,30,31,32,33]. In addition, the pharmacological activities of *A. scholaris*, particularly anti-lung cancer activity, have not yet been fully explained. Previously, the main triterpenoids in leaves of *A. scholaris* were identified by HPLC and LC/MS/MS [31]. Seven triterpenoid peaks were identified as cylicodiscic acid (7.7%), betulin (5.8%), betulinic acid (5.4%), oleanolic acid (15.1%), ursolic acid (23.6%), cycloeucalenol (10.3%), and α-amyrin acetate (6.5%), respectively. They found that the portion of triterpenoids showed a high anti-proliferative activity in A549 cells with IC_50_ values of 9.3 μg/mL. Several papers reported that ursolic acid possesses strong anti-cancer activity against several cancers of the prostate, breast, lung, pancreas, and bladder [34,35,36,37]. Ursolic acid had been isolated from *R. formosanum*, an endemic species distributed widely in Taiwan [38]. Way et al. focused on the antineoplastic effect of ursolic acid on NSCLC cells, and found that ursolic acid activated AMP-activated protein kinase (AMPK), and then inhibited the mTOR pathway, which controls protein synthesis and cell growth. These findings suggested that ursolic acid is a potent anti-cancer agent. In this study, we have investigated the chemical constituents and anti-proliferative activity of *A. scholaris* against NSCLC cells. We found that the major components with anti-proliferative activity in the leaves of *A. scholaris* were ursolic acid and betulinic acid. Oleanolic acid did not possess any anti-proliferative activity against A549 cells in this study. Moreover, compound **5** (2β,3β,28-lup-20(29)-ene-triol) also showed anti-proliferative activity against A549 cells. Our data suggest that not only ursolic acid, but also betulinic acid, is a potent anti-cancer agent. Previously studies have demonstrated that betulinic acid has anti-proliferative properties in vitro and in vivo [39,40]. Betulinic acid was able to trigger the mitochondrial pathway of apoptosis to induce apoptotic cell death in cancer cells [41,42]. In mice, pharmacokinetic studies demonstrated that betulinic acid was well absorbed and distributed within the melanoma xenografts [43]. In addition, normal cells and tissue are relatively resistant to betulinic acid, pointing to a therapeutic usage [44]. Moreover, betulinic acid is being developed by a large network of clinical trial groups supported by the U.S. National Cancer Institute [45]. Therefore, it is tempting to propose that *A. scholaris* could be developed as an anti-cancer agent for NSCLC. 

Although sterols isolated in this study exhibited no cytotoxic effects on NSCLC cells, the ability of sterols in clinical trials to block cholesterol absorption sites in the human intestine. It is worth investigating whether sterols could help reduce cholesterol absorption in humans, especially these first isolated sterols from *A. scholaris*, including poriferasterol, epicampesterol, 6β-hydroxy-4-stigmasten-3-one, and ergosta-7,22-diene-3β,5α,6β-triol. In conclusion, the ability of ursolic acid, betulinic acid, betulin, and 2β,3β,28-lup-20(29)-ene-triol to inhibit the proliferative activity of NSCLC can constitute a valuable group of therapeutic agents in the future.

## 4. Materials and Methods 

### 4.1. General Procedures

The NMR spectra, including ^1^H (600 MHz), ^13^C (150 MHz), DEPT (150 MHz), and 2D (^1^H-^1^H COSY, HSQC, and HMBC), were recorded on an Agilent Technologies DD2 600 spectrometer (Agilent, Santa Clara, CA, USA). ESI-MS was measured on a Bruker Daltonics Esquire high capacity ion trap (HCT) mass spectrometer (Bruker Daltonic Inc., Billerica, MA, USA). Column chromatographies (CCs) were carried out on silica gel 60 (230–400 mesh, Merck, Darmstadt, Germany), LiChroprep RP-18 (40–63 μm, Merck, Darmstadt, Germany), and Sephadex LH-20 (Pharmacia, Uppsala, Sweden). Precoated silica gel plates (Kieselgel 60 F_254_, 0.25 mm, Merck, Darmstadt, Germany) and RP-18 plates (F_254_, Merck, Darmstadt, Germany) were used for analytical thin layer chromatography (TLC). The preparative HPLC was performed on a Hitachi HPLC system equipped with an L-2130 pump, and a Hitachi L-2420 UV-vis detector at 220 nm (Hitachi, Tokyo, Japan), using a Hibar Purospher RP-18e column (5 μm, 250 mm × 10 mm, Merck, Darmstadt, Germany).

### 4.2. Plant Materials

The leaves of *Alstonia scholaris* (L.) R. Br. were collected from an *A. scholaris* forest near Mingdao University (23°52′15.17″ N and 120°29′47.13″ E), Changhua County, Taiwan, in March 2011. The voucher specimen (2010-0118-Wang) was preserved in the Lab of Chemical Ecology, Research Center for Biodiversity, China Medical University. The plant species was identified by the Key Laboratory of the High Altitude Experimental Station within the Taiwan Endemic Species Research Institute. 

### 4.3. Isolation and Identification of Triterpenoids and Sterols

**Figure 3:**
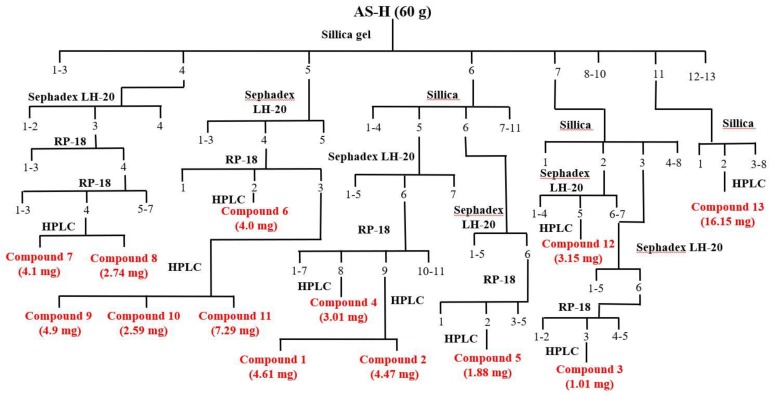
Purification flow chart of triterpenoids and sterols isolated from *A. scholaris*.

As shown in Figure 3, the anti-proliferative constitutes of the most effective fractions in the hexane portion (fraction Hex-4 to Hex-7) were isolated by using column chromatography to obtain 13 pure compounds.

*Ursolic acid* (**1**): White amorphous powder; ESI-MS *m*/*z* 479.3 [M + Na]^+^ (Calcd for C_30_H_48_O_3_: 456.3); ^1^H-NMR spectrum (600 MHz, CDCl_3_): δ 5.26 (1H, s, H-12), 3.23 (1H, dd, *J* = 10.7, 4.4 Hz, H-3), 1.08 (3H, s, Me-27), 0.99, 0.95, 0.93, 0.87, 0.82, 0.79 (Me-23, Me-30, Me-25, Me-29, Me-26, Me-24). ^13^C-NMR (150 MHz, CDCl_3_): δ C: 39.0 (C-1); 28.0 (C-2); 78.0 (C-3); 39.4 (C-4); 55.7 (C-5); 18.6 (C-6); 33.5 (C-7); 39.9 (C-8); 47.9 (C-9); 37.4 (C-10); 23.5 (C-11); 125.5 (C-12); 139.2 (C-13); 42.4 (C-14); 28.6 (C-15); 24.8 (C-16); 47.9 (C-17); 53.4 (C-18); 39.3 (C-19); 39.2 (C-20); 31.0 (C-21); 37.2 (C-22); 28.7 (C-23); 16.5 (C-24); 15.6 (C-25); 17.4 (C-26); 23.8 (C-27); 179.5 (C-28); 17.4 (C-29); 21.3 (C-30).

*Oleanolic acid* (**2**): White amorphous powder; ESI-MS *m*/*z* 479.3 [M + Na]^+^ (Calcd for C_30_H_48_O_3_: 456.3); ^1^H-NMR spectrum (600 MHz, CDCl_3_): δ 5.27 (1H, s, H-12), 3.22 (1H, dd, *J* = 10.6, 4.7 Hz, H-3), 2.82 (1H, dd, *J* = 13.5, 3.7 Hz, H-18), 1.13 (3H, s, Me-27), 0.98, 0.92, 0.91, 0.90, 0.77, 0.75 (Me-23, Me-26, Me-30, Me-24, Me-29, Me-25). ^13^C-NMR (150 MHz, CDCl_3_): δ C: 38.8 (C-1); 28.0 (C-2); 78.0 (C-3); 39.3 (C-4); 55.7 (C-5); 18.7 (C-6); 33.2 (C-7); 39.6 (C-8); 48.0 (C-9); 37.3 (C-10); 23.6 (C-11); 122.4 (C-12); 144.7 (C-13); 42.1 (C-14); 28.2 (C-15); 23.7 (C-16); 46.6 (C-17); 41.9 (C-18); 46.4 (C-19); 30.9 (C-20); 34.2 (C-21); 33.2 (C-22); 28.7 (C-23); 16.5 (C-24); 15.6 (C-25); 17.5 (C-26); 26.1 (C-27); 179.8 (C-28); 33.2 (C-29); 23.7 (C-30).

*Betulinic acid* (**3**): White crystal; ESI-MS *m*/*z* 455.3 [M − H]^−^ (Calcd for C_30_H_48_O_3_: 456.3); ^1^H-NMR spectrum (600 MHz, CDCl_3_): δ 4.74 (1H, brs, Hβ-29), 4.61 (1H, brs, Hα-29), 3.19 (1H, dd, *J* = 11.5, 4.8 Hz, H-3), 3.00 (1H, m, H-19), 1.69, 0.97, 0.96, 0.94, 0.82, 0.75 (Me-30, Me-27, Me-23, Me-26, Me-25, Me-24), 0.68 (1H, d, *J* = 9.0 Hz, H-5). ^13^C-NMR (150 MHz, CDCl_3_): δ C: 38.7 (C-1); 27.3 (C-2); 79.0 (C-3); 38.8 (C-4); 55.3 (C-5); 18.2 (C-6); 34.3 (C-7); 40.6 (C-8); 50.5 (C-9); 37.0 (C-10); 20.8 (C-11); 25.4 (C-12); 38.3 (C-13); 42.4 (C-14); 30.5 (C-15); 32.1 (C-16); 56.2 (C-17); 46.8 (C-18); 49.2 (C-19); 150.3 (C-20); 29.6 (C-21); 37.2 (C-22); 27.9 (C-23); 15.3 (C-24); 16.1 (C-25); 16.0 (C-26); 14.6 (C-27); 179.5 (C-28); 109.6 (C-29); 19.3 (C-30).

*Betulin* (**4**): White amorphous powder; ESI-MS *m*/*z* 465.3 [M + Na]^+^ (Calcd for C_30_H_50_O_2_: 442.3); ^1^H-NMR spectrum (600 MHz, CDCl_3_): δ 4.69 (1H, brs, Hβ-29), 4.58 (1H, brs, Hα-29), 3.80 (1H, d, *J* = 10.8 Hz, Hβ-28), 3.33 (1H, d, *J* = 10.8 Hz, Hα-28), 3.19 (1H, dd, *J* = 11.5, 4.7 Hz, H-3), 2.38 (1H, m, H-19), 1.68, 1.02, 0.98, 0.97, 0.82, 0.76 (Me-30, Me-26, Me-27, Me-23, Me-25, Me-24), 0.68 (1H, d, *J* = 9.6 Hz, H-5). ^13^C-NMR (150 MHz, CDCl_3_): δ C: 38.6 (C-1); 27.3 (C-2); 78.9 (C-3); 38.8 (C-4); 55.2 (C-5); 18.2 (C-6); 34.2 (C-7); 40.9 (C-8); 50.3 (C-9); 37.1 (C-10); 20.8 (C-11); 25.1 (C-12); 37.2 (C-13); 42.7 (C-14); 27.0 (C-15); 29.1 (C-16); 47.7 (C-17); 48.7 (C-18); 47.7 (C-19); 150.4 (C-20); 29.7 (C-21); 33.9 (C-22); 27.9 (C-23); 15.3 (C-24); 16.0 (C-25); 15.9 (C-26); 14.7 (C-27); 60.5 (C-28); 109.6 (C-29); 19.0 (C-30).

*2*β*,3*β*,28-lup-20(29)-ene-triol* (**5**): White solid; ESI-MS *m*/*z* 481.4 [M + Na]^+^ (Calcd. for C_30_H_50_O_3_: 458.3); ^1^H-NMR spectrum (600 MHz, CDCl_3_) and ^13^C-NMR (150 MHz, CDCl_3_) are listed in Table 1.

*Lupeol* (**6**): White amorphous powder; ESI-MS *m*/*z* 449.4 [M + Na]^+^ (Calcd for C_30_H_50_O: 426.3); ^1^H-NMR spectrum (600 MHz, CDCl_3_): δ 4.69 (1H, brs, Hβ-29), 4.57 (1H, brs, Hα-29), 3.20 (1H, m, H-3), 2.38 (1H, m, H-19), 1.68 (3H, s, Me-30), 1.03, 0.97, 0.95, 0.83, 0.79, 0.76 (Me-26, Me-27, Me-23, Me-25, Me-28, Me-24), 0.68 (1H, d, *J* = 9.6 Hz, H-5). ^13^C-NMR spectrum (150 MHz, CDCl_3_): δ C: 38.0 (C-1), 25.0 (C-2), 78.9 (C-3), 38.6 (C-4), 55.2 (C-5), 18.2 (C-6), 34.2 (C-7), 40.7 (C-8), 50.3 (C-9), 37.1 (C-10), 20.8 (C-11), 27.4 (C-12), 38.8 (C-13), 42.7 (C-14), 27.9 (C-15), 35.5 (C-16), 42.9 (C-17), 48.2 (C-18), 47.9 (C-19), 150.9 (C-20), 29.8 (C-21), 39.9 (C-22), 29.6 (C-23), 15.3 (C-24), 16.1 (C-25), 15.9 (C-26), 14.5 (C-27), 17.9 (C-28), 109.3 (C-29), 19.2 (C-30). 

β*-Amyrin* (**7**): Colorless solid; ESI-MS *m*/*z* 449.6 [M + Na]^+^ (Calcd for C_30_H_50_O: 426.3); ^1^H-NMR spectrum (600 MHz, CDCl_3_): δ 5.18 (1H, t, *J* = 3.6 Hz, H-12), 3.23 (1H, dd, *J* = 10.4, 4.8 Hz, H-3), 1.94 (1H, dd, *J* = 14.4, 4.8 Hz, H-18), 1.56 (1H, dd, *J* = 7.8, 1.8 Hz, H-9), 1.13 (3H, s, Me-27), 0.99, 0.96, 0.93, 0.88, 0.87, 0.83, 0.79 (Me-23, Me-26, Me-25, Me-29, Me-30, Me-28, Me-24), 0.74 (1H, dd, *J* = 12.0, 1.2 Hz, H-5). ^13^C-NMR spectrum (150 MHz, CDCl_3_): δ C: 38.5 (C-1), 27.2 (C-2), 79.0 (C-3), 38.7 (C-4), 55.1 (C-5), 18.3 (C-6), 32.6 (C-7), 39.7 (C-8), 47.6 (C-9), 36.9 (C-10), 23.5 (C-11), 121.7 (C-12), 145.2 (C-13), 41.7 (C-14), 26.1 (C-15), 26.9 (C-16), 32.4 (C-17), 47.2 (C-18), 46.8 (C-19), 31.1 (C-20), 34.7 (C-21), 37.1 (C-22), 28.0 (C-23), 15.5 (C-24), 15.5 (C-25), 16.7 (C-26), 25.9 (C-27), 28.3 (C-28), 33.3 (C-29), 23.6 (C-30).

α*-Amyrin* (**8**): Colorless solid; ESI-MS *m*/*z* 449.6 [M + Na]^+^ (Calcd for C_30_H_50_O: 426.3); ^1^H-NMR spectrum (600 MHz, CDCl_3_): δ 5.13 (1H, t, *J* = 3.6 Hz, H-12), 3.30 (1H, dd, *J* = 11.4, 5.4 Hz, H-3), 1.99 (2H, td, *J* = 13.5, 4.8 Hz, H-15), 1.84 (2H, td, *J* = 13.6, 4.9 Hz, H-16), 0.92 (3H, d, *J* = 6.0 Hz, Me-30), 0.78 (3H, d, *J* = 4.8 Hz, Me-29), 1.07, 1.01, 1.00, 0.95, 0.80, 0.79 (Me-27, Me-26, Me-23, Me-24, Me-28, Me-25), 0.74 (1H, dd, *J* = 12.0, 1.2 Hz, H-5). ^13^C-NMR spectrum (150 MHz, CDCl_3_): δ C: 38.7 (C-1), 28.0 (C-2), 79.0 (C-3), 38.7 (C-4), 55.1 (C-5), 18.3 (C-6), 32.9 (C-7), 39.9 (C-8), 47.7 (C-9), 36.8 (C-10), 23.3 (C-11), 124.4 (C-12), 139.5 (C-13), 42.0 (C-14), 27.2 (C-15), 26.6 (C-16), 33.7 (C-17), 59.0 (C-18), 39.6 (C-19), 39.6 (C-20), 31.2 (C-21), 41.5 (C-22), 28.1 (C-23), 15.6 (C-24), 15.6 (C-25), 16.8 (C-26), 23.2 (C-27), 28.7 (C-28), 17.4 (C-29), 21.4 (C-30).

*Poriferasterol* (**9**): White amorphous powder; EI-MS *m*/*z* 412.4 [M]^+^ (Calcd for C_29_H_48_O: 412.4); ^1^H-NMR spectrum (600 MHz, CDCl_3_): δ 5.35 (1H, t, *J* = 5.3, H-6), 5.15 (1H, m, H-22), 5.01 (1H, m, H-23), 3.52 (1H, m, H-3), 2.30 (1H, dd, *J* = 13, 5.1, H-4β), 2.25 (1H, dd, *J* = 11.4, 5.3, H-4α), 1.01 (3H, d, *J* = 6.6, Me-21), 1.01, 0.85, 0.82, 0.80, 0.70 (Me-19, Me-28, Me-26, Me-29, Me-18). ^13^C-NMR (150 MHz, CDCl_3_): δ C: 37.2 (C-1); 31.6 (C-2); 71.7 (C-3); 42.2 (C-4); 140.7 (C-5); 121.7 (C-6); 31.8 (C-7); 31.8 (C-8); 50.1 (C-9); 36.4 (C-10); 21.2 (C-11); 39.6 (C-12); 42.1 (C-13); 55.9 (C-14); 24.3 (C-15); 28.9 (C-16); 56.8 (C-17); 12.0 (C-18); 19.3 (C-19); 40.5 (C-20); 21.0 (C-21); 138.3 (C-22); 129.2 (C-23); 51.2 (C-24); 25.4 (C-25); 12.2 (C-26); 28.9 (C-27); 21.0 (C-28); 18.9 (C-29).

*Epicampesterol* (**10**): Faint yellow powder; EI-MS *m*/*z* 400.3 [M]^+^ (Calcd for C_28_H_48_O: 400.3); ^1^H-NMR spectrum (600 MHz, CDCl_3_): δ 5.35 (1H, t, *J* = 5.4 Hz, H-6), 3.52 (1H, m, H-3), 2.30 (1H, dd, *J* = 13, 5.1, H-4β), 2.25 (1H, dd, *J* = 11.4, 5.3, H-4α), 0.92 (3H, d, *J* = 6.6, Me-21), 1.01, 0.85, 0.79, 0.78, 0.68 (Me-19, Me-28, Me-27, Me-25, Me-18). ^13^C-NMR (150 MHz, CDCl_3_): δ C: 37.2 (C-1); 31.6 (C-2); 71.8 (C-3); 42.2 (C-4); 140.7 (C-5); 121.7 (C-6); 31.9 (C-7); 31.9 (C-8); 50.1 (C-9); 36.5 (C-10); 21.0 (C-11); 39.7 (C-12); 42.2 (C-13); 56.7 (C-14); 24.2 (C-15); 28.1 (C-16); 55.9 (C-17); 11.8 (C-18); 19.4 (C-19); 36.1 (C-20); 18.9 (C-21); 33.7 (C-22); 32.4 (C-23); 39.0 (C-24); 15.4 (C-25); 31.4 (C-26); 17.6 (C-27); 20.5 (C-28). 

β*-sitosterol* (**11**): White waxy powders; ESI-MS *m*/*z* 469.3 [M + Na]^+^ (Calcd for C_29_H_50_O_3_: 456.3); ^1^H-NMR spectrum (600 MHz, CDCl_3_): δ 5.35 (1H, t, *J* = 5.4 Hz, H-6), 3.52 (1H, m, H-3), 2.30 (1H, dd, *J* = 13, 5.1, H-4β), 2.25 (1H, dd, *J* = 11.4, 5.3, H-4α), 0.92 (3H, d, *J* = 6.6, Me-21), 1.01, 0.84, 0.83, 0.81, 0.68 (Me-19, Me-26, Me-28, Me-29, Me-18). ^13^C-NMR (150 MHz, CDCl_3_): δ C: 37.2 (C-1); 31.6 (C-2); 71.8 (C-3); 42.2 (C-4); 140.7 (C-5); 121.7 (C-6); 31.8 (C-7); 31.8 (C-8); 50.1 (C-9); 36.4 (C-10); 21.0 (C-11); 39.7 (C-12); 42.2 (C-13); 56.7 (C-14); 24.2 (C-15); 28.2 (C-16); 56.0 (C-17); 36.1 (C-20); 19.0 (C-21); 33.9 (C-22); 26.0 (C-23); 45.8 (C-24); 23.0 (C-25); 11.9 (C-26); 29.1 (C-27); 19.8 (C-28); 19.3 (C-29); 18.7 (C-19); 11.8 (C-18).

*6*β*-Hydroxy-4-stigmasten-3-one* (**12**): White amorphous powder; EI-MS *m*/*z* 428.4 [M]^+^ (Calcd for C_29_H_48_O_2_: 428.4); ^1^H-NMR spectrum (600 MHz, CDCl_3_): δ 5.83 (1H, s, H-4), 4.34 (1H, brs, H-6), 2.52 (1H, dd, *J* = 15.1, 4.9, H-2β), 2.39 (1H, dd, *J* = 15.1, 3.1, H-2α), 1.38 (3H, s, Me-19), 0.93, 0.86, 0.84, 0.82, 0.75 (Me-21, Me-27, Me-24, Me-25, Me-18). ^13^C-NMR (150 MHz, CDCl_3_): δ C: 37.0 (C-1); 34.2 (C-2); 200.3 (C-3); 126.4 (C-4); 168.6 (C-5); 73.2 (C-6); 38.5 (C-7); 29.7 (C-8); 53.6 (C-9); 37.9 (C-10); 20.9 (C-11); 39.5 (C-12); 42.4 (C-13); 55.8 (C-14); 24.1 (C-15); 28.1 (C-16); 56.0 (C-17); 12.0 (C-18); 19.5 (C-19); 36.1 (C-20); 18.7 (C-21); 33.8 (C-22); 26.0 (C-23); 45.8 (C-24); 29.1 (C-25); 19.8 (C-26); 19.0 (C-27); 23.0 (C-28); 12.0 (C-29). 

*Ergosta-7,22-diene-3*β*,5*α*,6*β*-triol* (**13**): White needles; ESI-MS *m*/*z* 453.4 [M + Na]^+^ (Calcd for C_28_H_46_O_3_: 430.3); ^1^H-NMR spectrum (600 MHz, CDCl_3_): δ 5.35 (1H, d, *J* = 3.0 Hz, H-7), 5.23 (1H, dd, *J* = 15.6, 7.8 Hz, H-23), 5.16 (1H, dd, *J* = 15.6, 8.4 Hz, H-22), 4.08 (1H, m, H-3), 3.62 (1H, d, *J* = 5.4 Hz, H-6), 1.08 (3H, s, Me-19), 1.02, 0.91, 0.84, 0.82, 0.60 (Me-21, Me-26, Me-27, Me-28, Me-18). ^13^C-NMR (150 MHz, CDCl_3_): δ C: 32.9 (C-1); 30.8 (C-2); 67.7 (C-3); 39.4 (C-4); 75.9 (C-5); 73.6 (C-6); 117.4 (C-7); 144.0 (C-8); 43.4 (C-9); 37.1 (C-10); 22.0 (C-11); 39.1 (C-12); 43.7 (C-13); 54.7 (C-14); 22.8 (C-15); 27.9 (C-16); 55.9 (C-17); 12.3 (C-18); 18.8 (C-19); 40.4 (C-20); 21.1 (C-21); 135.3 (C-22); 132.1 (C-23); 42.7 (C-24); 33.0 (C-25); 17.5 (C-26); 19.9 (C-27); 19.6 (C-28).

### 4.4. Anti-Proliferative Activity

Antiproliferation activity was determined against A549 cells (human lung adenocarcinoma cell line) using the MTT assay (Promega, Fitchburg, WI, USA). Briefly, the A549 cell line was cultured in Dulbecco’s modified eagle medium (DMEM) supplemented with 10% fetal bovine serum and antibiotics (100 U/mL of penicillin and 100 μg/mL of streptomycin). This assay is based on the cleavage of the MTT to purple formazan crystals by metabolically active cells. MTT assay was done as described previously [38]. Briefly, the A549 cells were inoculated into a 96-well culture plate (1 × 10^4^ cells/well) and treated with tested compounds in different concentrations at 37 °C for 48 h. After removing the medium from each well, 100 μL of MTT (500 μg/mL) was added to each well, and the plate was incubated at 37 °C for 1 h. When purple precipitate was clearly visible under the microscope, 80 μL of DMSO was added to each well. The plate was incubated in the dark for 1 h at room temperature. The spectrophotometric absorbance of the samples was detected by using an ELISA reader (SpectraMax M5e, Molecular Devices LLC, Sunnyvale, CA, USA) at 570 nm. The cell viability was calculated as the percentage of cell survival after the treatment. All measurements were performed in triplicate. 

## 5. Conclusions

Eight triterpenoids and five sterols have been isolated from the hexane portion of *A. scholaris* leaves. 2β,3β,28-lup-20(29)-ene-triol was the first reported natural product isolated from the plant. In addition, poriferasterol, epicampesterol, 6β-Hydroxy-4-stigmasten-3-one, and ergosta-7,22-diene-3β,5α,6β-triol were also isolated from *A. scholaris* for the first time. The ability of ursolic acid, betulinic acid, betulin, and 2β,3β,28-lup-20(29)-ene-triol to inhibit the NSCLC proliferative activity can constitute a valuable group of therapeutic agents in the future.

## Figures and Tables

**Table 1:** The ^1^H- and ^13^C-NMR spectral data of compound **5** and 2β,3β,28-lup-20(29)-ene-triol [29].

Compound	5		2β,3β,28-lup-20(29)-ene-triol	
Position	^1^H	^13^C	^1^H	^13^C
**1**	2.15, 1.09	44.4		44.5
**2**	4.09 (dd, *J* = 3.6, 6.6)	71.1	4.08 (brs)	71.2
**3**	3.19 (d, *J* = 4.2)	78.4	3.19 (brs)	78.5
**4**		38.1		38.2
**5**	0.77 (d, *J* = 9.5)	55.2		55.3
**6**	1.56	18.2		18.1
**7**	1.41	34.1		34.2
**8**		41		41.1
**9**	1.24	50.8		50.9
**10**		36.8		36.9
**11**	1.45	20.9		21.0
**12**	1.65, 1.05	25.2		25.3
**13**	1.65	37.2		37.3
**14**		42.8		42.9
**15**	1.71, 1.05	26.9		27.0
**16**	1.93, 1.21	29.1		29.2
**17**		47.7		47.8
**18**	1.58	48.7		48.8
**19**	2.39 m	47.7		47.8
**20**		150.4		150.4
**21**		29.7		29.8
**22**	1.86, 1.04	33.9		34.0
**23**	0.99 s	29.5	0.99 s	29.6
**24**	0.98 s	17.1	0.98 s	17.1
**25**	1.14 s	17.0	1.14 s	17.1
**26**	1.04 s	15.9	1.04 s	16.0
**27**	0.97 s	14.7	0.97 s	14.7
**28**	3.80, 3.33 (d, *J* = 10.8)	60.5	3.80, 3.33 (d, *J* = 10.8)	60.6
**29**	4.69, 4.59	109.6	4.69, 4.58	109.7
**30**	1.68 s	19.2	1.68 s	19.1

**Table 2:** The inhibitory concentration of (IC_50_) of triterpenoids on non-small-cell lung cancer (NSCLC).

Compound	NSCLC (A549 Cell Line)	
	IC_50_ (μM)	S.E. ^+^
Ursolic acid (**1**)	39.8	0.09
Oleanolic acid (**2**)	>400	-
Betulinic acid (**3**)	40.1	0.51
Betulin (**4**)	240.5	4.04
2β,3β,28-lup-20(29)-ene-triol (**5**)	172.6	0.44
Lupeol (**6**)	>400	-
β-amyrin (**7**)	>400	-
α-amyrin (**8**)	>400	-

^+^ S.E.: standard error.

## Abbreviations

AMPK	AMP-activated protein kinase
COSY	Correlation spectroscopy
DEPT	Distortionless Enhancement by Polarization Transfer
DMEM	Dulbecco’s Modified Eagle Medium
HMBC	Heteronuclear Multiple Bond Correlation
HMQC	Heteronuclear Multiple-Quantum Correlation
mTOR	Mammalian target of rapamycin
MTT	3-(4,5-Dimethylthiazol-2-yl)-2,5-diphenyltetrazolium bromide
MRSA	Methicillin-resistant *Staphylococcus aureus*
NMR	Nuclear Magnetic Resonance
NSCLC	Non-Small Cell Lung Cancer
SCLC	Small Cell Lung Cancer
TLC	Thin layer chromatography
